# Progress in mass spectrometry approaches to profiling protein–protein interactions in the studies of the innate immune system

**DOI:** 10.1007/s42485-024-00156-6

**Published:** 2024-06-28

**Authors:** Doeun Kim, Aleksandra Nita‑Lazar

**Affiliations:** 1 Functional Cellular Networks Section, Laboratory of Immune System Biology, National Institute of Allergy and Infectious Diseases, National Institutes of Health, Bethesda, MD 20892-1892, USA

**Keywords:** Protein–protein interactions, Affinity purification mass spectrometry (AP-MS), Proximity labeling mass spectrometry (PL-MS), Cross-linking mass spectrometry (XL-MS), Size exclusion chromatography coupled with mass spectrometry (SEC-MS), Limited proteolysis-coupled mass spectrometry (LiP-MS), Thermal proteome profiling (TPP)

## Abstract

Understanding protein–protein interactions (PPIs) is pivotal for deciphering the intricacies of biological processes. Dysregulation of PPIs underlies a spectrum of diseases, including cancer, neurodegenerative disorders, and autoimmune conditions, highlighting the imperative of investigating these interactions for therapeutic advancements. This review delves into the realm of mass spectrometry-based techniques for elucidating PPIs and their profound implications in biological research. Mass spectrometry in the PPI research field not only facilitates the evaluation of protein–protein interaction modulators but also discovers unclear molecular mechanisms and sheds light on both on- and off-target effects, thus aiding in drug development. Our discussion navigates through six pivotal techniques: affinity purification mass spectrometry (AP-MS), proximity labeling mass spectrometry (PL-MS), cross-linking mass spectrometry (XL-MS), size exclusion chromatography coupled with mass spectrometry (SEC-MS), limited proteolysis-coupled mass spectrometry (LiP-MS), and thermal proteome profiling (TPP).

## Introduction

Biological molecules such as DNA, RNA, proteins, and metabolites play an important role in regulating biological systems. The outcomes of biological processes are determined by the interactions between these molecules. Protein–protein interactions (PPIs) are essential for modulating protein dynamics and characteristics (Arancibia et al. 2007; Jensen and Thomsen 2012; Koyama et al. 2008; Rebsamen et al. 2013; Rual et al. 2005; Thompson et al. 2011). An imbalance in PPIs is linked to a wide range of diseases such as cancer (Jiang et al. 2013; Kanhaiya et al. 2017; Kim et al. 2021; Li et al. 2012), neurodegenerative disorders (George et al. 2019; Karbalaei et al. 2018; Sinsky et al. 2021; Tomkins and Manzoni 2021; Tracy et al. 2022), and innate immune system aberrations (Corleis and Dorhoi 2020; Hornung et al. 2009; Li et al. 2011; Moncrieffe et al. 2020; Muruve et al. 2008; Pleska et al. 2018). This emphasizes the necessity to explore these interactions for potential therapies. In the context of the innate immune system, PPIs are crucial for mediating host–pathogen interactions, regulating inflammatory responses, and controlling immune cell activation. Comprehending the complexities of these interactions is essential for understanding the mechanisms behind innate immune signaling and discovering new therapeutic targets for infectious and inflammatory diseases (Ciuffa et al. 2022; Meng et al. 2021; Pereira and Gazzinelli 2023; Rajpoot et al. 2022; Van Quickelberghe et al. 2018). Studying PPIs is crucial for obtaining an in-depth understanding of biology. Despite the critical significance of PPIs, advancements in this field have been hindered by the requirement for an extensive knowledge of proteins and protein complexes.

**Fig. 1:**
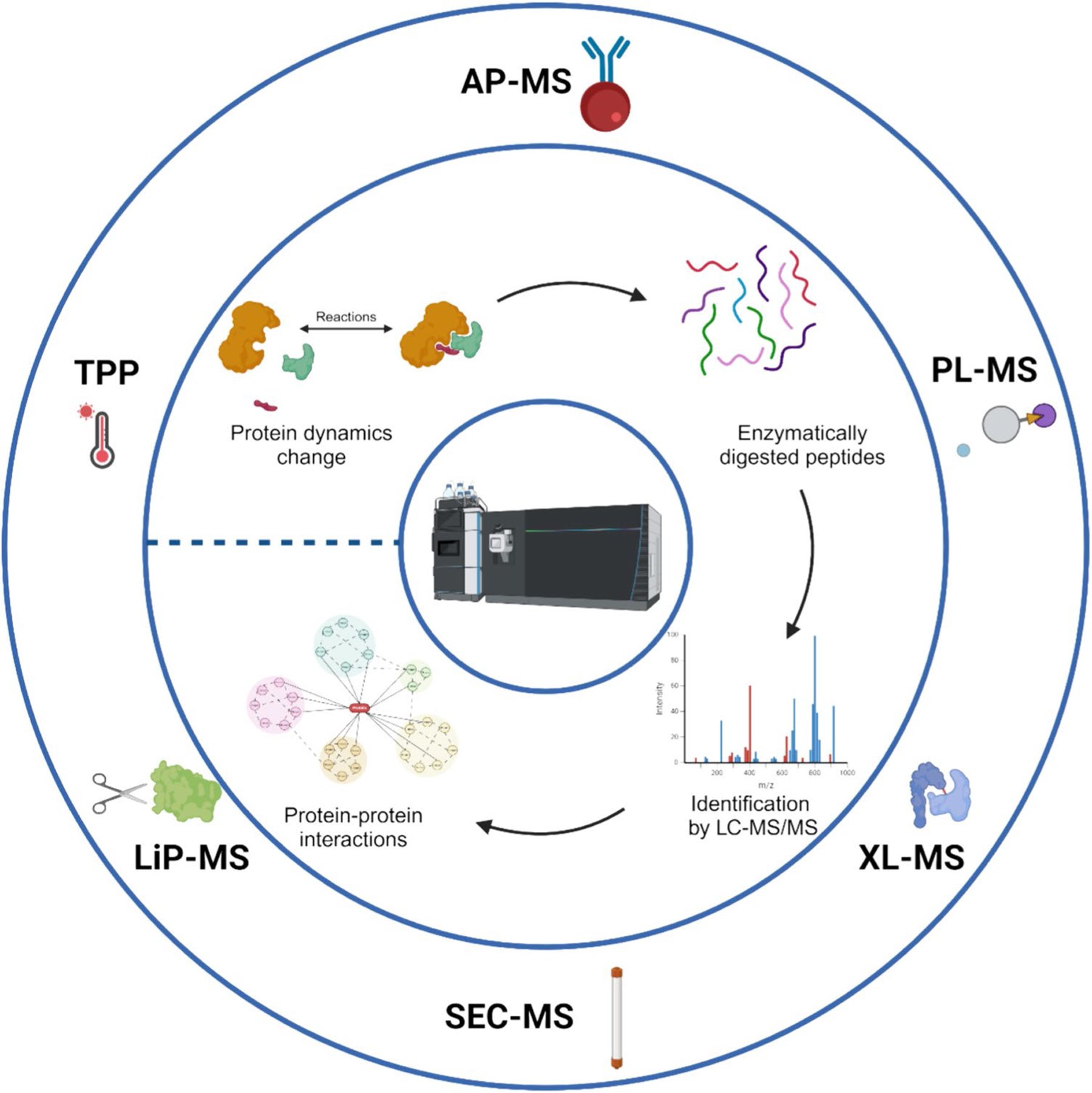
Overview of mass spectrometry-based techniques for protein–protein interactions (PPIs) study

Lately, mass spectrometry (MS)-based methods have emerged as effective tools for PPIs with outstanding sensitivity and specificity. These methods allow for the systematic evaluation of protein complexes and the discovery of interacting counterparts in intricate signaling networks. By combining MS-based proteomics with techniques including immunoprecipitation, cross-linking, limited proteolysis, and thermal profiling, researchers can improve their understanding of the dynamics and control of signaling pathways (Fig. 1).

This review offers an extensive overview of mass spectrometry-based methods used to investigate PPIs with the focus on innate immunity. We introduce the principles, methodologies, recent advancements, applications, advantages, and limitations of five main techniques: affinity purification mass spectrometry (AP-MS), proximity labeling mass spectrometry (PL-MS; BioID and its derivatives), cross-linking mass spectrometry (XL-MS), size exclusion chromatography coupled with mass spectrometry (SEC-MS), limited proteolysis-coupled mass spectrometry (LiP-MS), and thermal proteome profiling (TPP) to characterize PPIs resulting in biological processes to understand molecular mechanisms in innate immune system.

## Affinity purification mass spectrometry (AP‑MS)

**Fig. 2:**
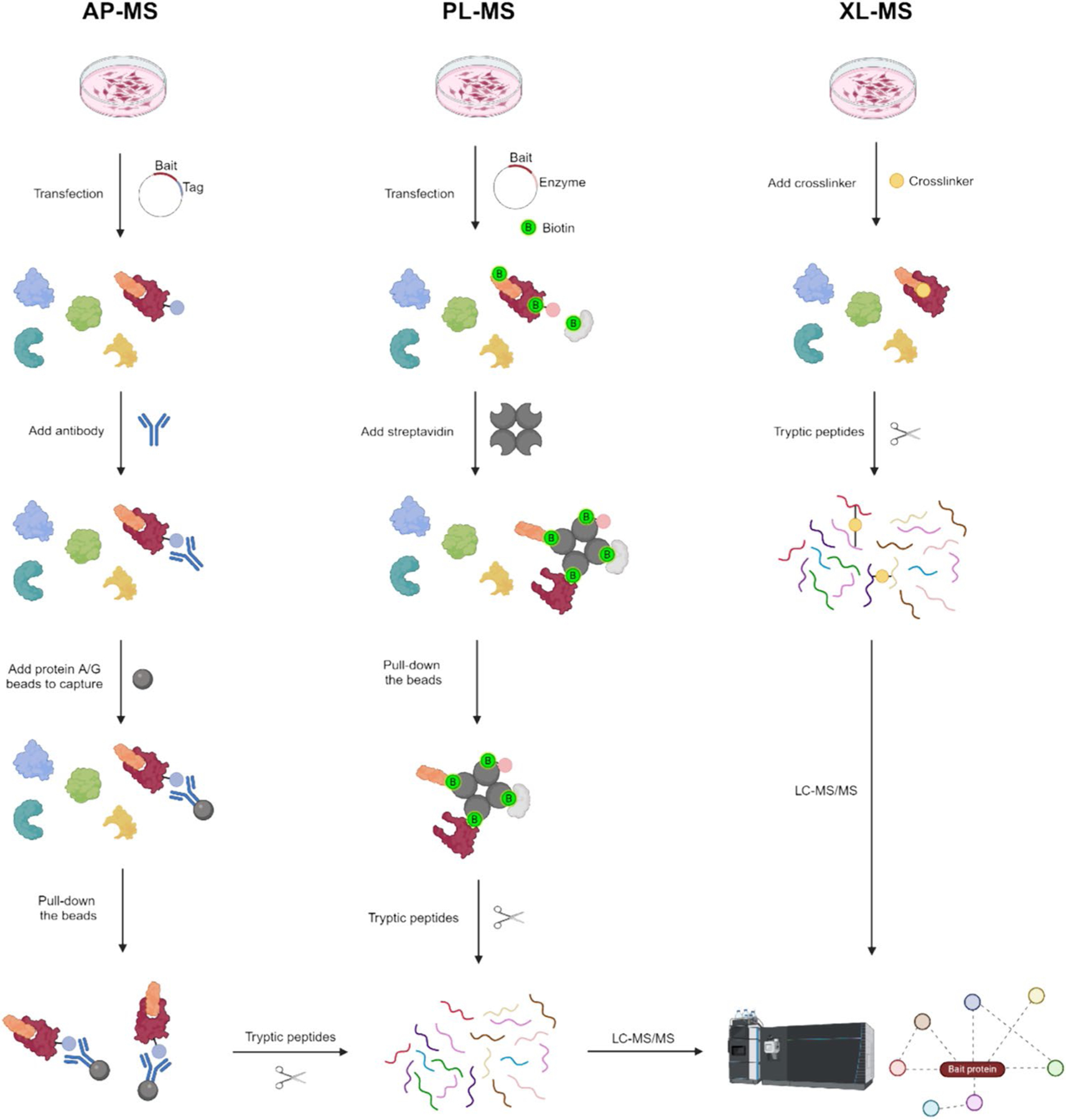
Workflows for affinity purification mass spectrometry (AP-MS), proximity labeling mass spectrometry (PL-MS), and cross-linking mass spectrometry (XL-MS)

AP-MS is a potent method used to discover and analyze PPIs in biological systems. This method merges the specificity of affinity purification with the sensitivity and accuracy of mass spectrometry to reveal the structural components of protein complexes (Bauer and Kuster 2003; Gingras et al. 2005). Numerous ligands, such as chemicals (Kool et al. 2011; Raida 2011; Rix and Superti-Furga 2009) and biomolecules (Busby et al. 2020; Butter et al. 2009; Faoro and Ataide 2014; Slobodin and Gerst 2011; Tsai et al. 2011), can be used for affinity purification. Following the purification of biological samples, proteins bound to ligands can be separated to simplify the sample or prepared for examination right away with mass spectrometry (Fig. 2). AP-MS has become a fundamental technique for analyzing PPI networks, offering important knowledge about cellular signaling pathways, protein complex formation, and disease processes.

AP-MS is based on isolating a specific protein or protein complex from a complex biological material and then identifying and quantifying the proteins that interact with binding partners using mass spectrometry. Affinity purification is commonly done by utilizing antibodies that target the bait protein or using affinity tags fused to the bait protein. This method allows for the separation of proteins that interact with the bait from cell lysates or tissue extracts (Dunham et al. 2012). AP-MS technique has been used to discover new protein–protein interactions that occur under the most significant physiological environments, whereas co-immunoprecipitation (Co-IP) is utilized to investigate the interaction between a known protein and its partners that are expressed in their native physiological conditions. This method uses conjugation with protein A/G beads and target protein-specific antibodies for immunoprecipitation to indirectly capture the proteins associated with the target protein (Lin and Lai 2017; Munoz and Castellano 2018). The interaction between the target protein and the binding protein is confirmed by western blotting. Co-IP is frequently used to identify the molecular pathways involved in inflammation, particularly in the study of the regulation of immunometabolism (Guo et al. 2021; Hu et al. 2021; McGauran et al. 2020), the activation of inflammasomes (Duan et al. 2020; Guan et al. 2021; Niu et al. 2021; Yang et al. 2015), and the networks of Toll-like receptors (Brikos et al. 2007; Kim et al. 2019; Lee et al. 2016; Shahinuzzaman et al. 2022). Both the AP-MS and Co-IP approaches rely on the capacity of the interaction partners to be captured along with the protein of interest.

AP-MS is advantageous because it can capture stable and transient protein interactions, offering a complete view of the protein interactome in specific experimental conditions. AP-MS allows for the purification of protein complexes in their natural shape using antibodies or tags that target the bait protein, thus maintaining the integrity of PPIs. Various adaptations of AP-MS have been created to improve its sensitivity, specificity, and flexibility. Tandem affinity purification (TAP) involves using two different affinity tags in a sequence to purify a protein complex through multiple purification processes, enhancing the purity of isolated proteins (Bian et al. 2022; Burckstummer et al. 2006; Gregan et al. 2007; Kaja et al. 2023; Volkel et al. 2010). Epitope tagging has the huge benefit of being able to tag several proteins with the same epitope and purify them using the same method. Hence, the background contaminants need to be consistent across all purification procedures to enable effective control experiments. AP-MS has been extensively used in diverse biological research fields, such as the investigation of innate immune signaling pathways and host–pathogen interactions (DeBlasio et al. 2021; Gillen and Nita-Lazar 2017; Morwitzer et al. 2019; Terracciano et al. 2021; van Zuylen et al. 2012). This method allowed discoveries of new interactors and regulatory mechanisms involved in innate immune signaling. New modulators of the tumor necrosis factor (TNF)-α/NF-κB pathway have been identified (Bouwmeester et al. 2004) using the tandem affinity purification (TAP) strategy. The authors used an integrated approach, combining large-scale pathway mapping with loss-of-function analysis and created a physical and functional map of the pathway interactions. Stutz et al. identified the components of the NLRP3 inflammasome and the essential novel regulatory phosphorylation sites using AP-MS with FLAG-tagged NLRP3 as a bait (Stutz et al. 2017). A systematic AP-MS approach allowed for the detailed functional analysis of the TBK1/IKKi complex and the associated molecular network (Goncalves et al. 2011).

AP-MS is a potent method for detecting PPIs in complicated biological systems, although it has several limitations. The specificity of the procedure depends on the affinity tag or antibody used for purification, which may result in false positives from non-specific binding or cross-reactivity. Furthermore, AP-MS might not identify weak or transient interactions, and complex samples can mask actual interactions due to background noise. Purifying insoluble or membrane-bound proteins can be difficult, and analyzing the data necessitates advanced computational techniques. Validating interactions is essential but laborious, and AP-MS investigations can be expensive and resource-intensive, which restricts accessibility. Despite its challenges, AP-MS is still an invaluable and widely used tool for studying protein interactions in biological systems when combined with other techniques.

## Proximity labeling mass spectrometry (PL‑MS)

Proximity labeling mass spectrometry (PL-MS) has advanced the PPIs and spatial proximity in the biological environment (Fig. 2). The major enzyme classes applied for proximity labeling techniques include bacterial biotin ligase (BirA*) and peroxidase-based enzymes such as horseradish peroxidase (HRP) and ascorbate peroxidase (APEX). HRP and APEX proximity labeling depends on fusing the protein of interest with peroxidase-based enzymes. When hydrogen peroxide and biotin-phenol activate these enzymes, they produce short-lived biotin-phenoxyl radicals (Mortensen and Skibsted 1997). The radicals form covalent bonds with biotin atoms that are near proteins, with a specific focus on tyrosine, lysine, and histidine residues located within a range of 10–20 nm. Streptavidin affinity purification is used to concentrate biotinylated proteins, making it easier to identify and measure them by mass spectrometry analysis (Hung et al. 2014; Rhee et al. 2013).

HRP is a well-researched peroxidase enzyme that has been used for proximity labeling. Nevertheless, it showed low labeling efficiency in reducing conditions (Trinkle-Mulcahy 2019). Engineered APEX overcomes this restriction and can be genetically involved as a tag on desired bait proteins. Due to the rapid labeling capabilities of APEX, which are comparable in speed to numerous biological processes, this method is ideally adapted for studying protein interactions that are transient or subject to protein dynamic change. APEX labeling is effective in multiple subcellular locations due to its ability to function in reducing conditions, such as the cytosol (Martell et al. 2012).

BioID is an innovative approach used to characterize PPIs and spatial proximity within cells. BioID uses a proximity-based labeling strategy to capture transient or weak interactions around the bait protein, unlike typical affinity purification methods. This method involves using a fusion protein that includes the protein of interest and an adaptable biotin ligase enzyme like BirA* to facilitate the biotinylation of surrounding proteins. Streptavidin affinity purification isolates the biotinylated proteins for further mass spectrometry analysis. BirA* enzyme converts biotin into a more reactive form, which then combines with primary amines of adjacent proteins, leading to their covalent biotinylation. (Roux et al. 2018).

BioID’s application for the study of temporal protein complexes is restricted as it requires 12–24 h to acquire sufficient labeling signals via mass spectrometry, owing to its slow reaction kinetics. It may result in off-target labeling, high background, and limit the range of experiments suitable for BioID. Furthermore, BioID investigations are restricted to creating static interaction maps because of their temporality. To surpass this restriction, BioID2 was created by modifying the biotin ligase of *Aquifex aeolicus* through mutations (Kim et al. 2016). This small enzyme greatly reduces the interference with the fusion protein, resulting in enhanced targeting and localization to subcellular compartments (Kim et al. 2017). Nevertheless, it still takes more than 16 h to label. To reduce these experimental limitations, TurboID and miniTurbo were invented. TurboID has 15 mutations and miniTurbo has 13 mutations, and a deletion of the N-terminal domain. These enzymes have a strong affinity to biotin, enabling efficient labeling similar to BioID in less than 10 min. Split proximity labeling methods can be utilized for situations where conventional proximity labeling methodologies are unable to access the region of interest, such as organelle contact sites, to achieve higher targeted specificity. Split proximity labeling methods may also be utilized in situations where large protein fusions are intolerable due to the significantly reduced size of individual fragments. Split forms of APEX (Han et al. 2019), HRP (Heo et al. 2023; Martell et al. 2016), BioID (De Munter et al. 2017; Kwak et al. 2020; Ramirez et al. 2021; Schopp et al. 2017), and TurboID (Cho et al. 2020a, b; Cho et al. 2020a, b; Fujimoto et al. 2023; Schmitt et al. 2021; Shkel et al. 2022; Xu et al. 2021) have been created, where two nonfunctional parts can combine to generate a whole enzyme.

The utilization of PL-MS advanced the process of deciphering the structure and functioning of innate immune signaling networks. BioID was utilized to discover 111 proteins linked to caspase-1 during inflammasome activation in a cell-free system, pointing into the direction of p62/sequestosome1 protein as a regulator of caspase-1-induced inflammation (Jamilloux et al. 2018). A biotin environment scan identified specific interaction partners of the mitochondrial anchored protein ligase (MAPL) a component of MAVS (mitochondrial antiviral signaling) complex in the context of the Sendai virus infection, highlighting a critical role for MAPL and mitochondrial SUMOylation in the early phases of antiviral signaling (Doiron et al. 2017). May et al. used BioID to characterize the SARS-CoV2 virus–host interactome in human lung cancer-derived cells (May et al. 2022). The TurboID technique was utilized to gain a deep understanding of the molecular pathways involved in NLR immune receptor-mediated immunity (Zhang et al. 2019) and SARS-CoV2 suppression (Zhang et al. 2022). Through the process of elucidating the protein interactions that occur during infections, researchers could get a more comprehensive understanding of the host–pathogen interactions. This understanding may lead to the discovery of novel therapeutic targets. These state-of-the-art tools have emerged as a potent tool for understanding the structure and functioning of these signaling networks.

Nevertheless, APEX may lack the precision required for distinguishing direct interactions from proteins merely in proximity due to diffusion. Activating APEX with hydrogen peroxide may induce cellular stress responses and result in non-specific labeling, thereby compromising the accuracy of the outcomes. In addition, the technique might encounter challenges in certain cellular compartments, such as mitochondria, because of the presence of natural biotinylated proteins that could interfere with labeling or complicate analysis. Optimizing APEX labeling may be necessary for different cell types or experimental conditions, which might make its application more complex.

BioID poses challenges due to non-specific biotinylation, leading to false-positive identifications. To address this problem, negative controls and rigorous wash processes should be employed while purifying streptavidin. These approaches guarantee the reliability and precision of BioID-based studies, hence improving its usefulness in biological research.

## Cross‑linking mass spectrometry (XL‑MS)

XL-MS is a method that chemically links amino acid residues in protein complexes and then uses mass spectrometry to analyze and identify the cross-linked peptides, allowing for the interpretation of spatial proximities between residues. The structural information is obtained by detecting adjacent pairs of amino acids, which may include weak or transient interactions, that are connected by a chemical cross-linker of a particular length (Fig. 2). The distance restraint information acquired can help define the structure and positioning of subunits inside the complex and PPI-binding sites (Yu and Huang 2018). The cross-linking process is carried out under the native condition. Enzymatic digestion creates cross-linked peptides which are then enriched, analyzed by MS, and identified through database searching. Data analysis reveals the sequence assignment of cross-linked peptides and identifies the specific cross-linked amino acid residues. Integrative modeling strategies can utilize physical interaction data obtained by XL-MS to enhance structural biology and computational modeling investigations. Restraint information is gathered for proteins that are connected both internally and externally, and used to analyze the arrangement of multiple protein complexes (Shi et al. 2014) and interactions across the entire proteome (Weisbrod et al. 2013). Various cross-linkers are accessible and designed to interact with different amino acid side chains and distances between binding interfaces (Chakrabarty et al. 2020; Gutierrez et al. 2018, 2016; Kao et al. 2011). In addition, several methods have been created to enhance the detection and precision of XL- MS by enriching cross-linked peptides. (Chavez and Bruce 2019; Kaake et al. 2014; Leitner et al. 2012; Liu et al. 2020; Rinner et al. 2008; Tang et al. 2005).

XL-MS was used for innate immune signaling studies, especially to map host and pathogen PPIs. XL-MS enabled the identification of functional interactions between bacterial Toll-interleukin receptor (TIR)-domain containing proteins and mammalian receptor TIR domains, revealing differences in binding dynamics between different TIR domain containing proteins and allowing for the further analysis by NMR resulting in the first atomic model of the human TIR domain from IL-1R8 protein (Lee et al. 2022). Intact extracellular human cytomegalovirus virion interactions with host proteins were mapped using XL-MS providing insights into virion organization and revealing crucial PPIs (Bogdanow et al. 2023). XL-MS was used to characterize the interactions between adenovirus C5 hexon and human lactoferrin (hLF), pinpointing major contact sites in hLF and suggesting a mechanism in which the virus uses hLF for cellular entry (Dhillon et al. 2024).

XL-MS has various benefits for analyzing protein complexes, such as its capacity to reveal structure-related information on PPIs within their native cellular environment. XL-MS is capable of capturing transient or weak interactions that could be overlooked in typical biochemical tests, enabling an in-depth examination of dynamic protein complexes participating in signaling networks. Nevertheless, XL-MS has drawbacks such as complex data analysis, probable false-positive identifications, and low spatial resolution of cross-linkers. XL-MS experiments necessitate specialist knowledge in mass spectrometry, chemistry, and bioinformatics, thereby restricting their broad implementation in biological research labs.

## Size exclusion chromatography coupled with mass spectrometry (SEC‑MS)

Size exclusion chromatography (SEC), also referred to as gel filtration chromatography, is a traditional biochemical technique used to separate protein complexes without bias. This method proves valuable for examining and refining substantial biomolecules, such as proteins, nucleic acids, and polymers. SEC is a technique that separates molecules by their size or hydrodynamic volume. In this process, larger molecules are excluded from the pores of the stationary phase and elute first. Smaller molecules pass through the pores and so have a delayed elution (Fekete et al. 2014; Hartmann et al. 2004). The procedure of SEC-MS involves loading the sample into an SEC column, where the components are segregated based on their size. The eluent from the column is subsequently injected into the MS system, where each of the molecules is ionized and their mass-to-charge ratios are obtained (Havugimana et al. 2022; Skinnider and Foster 2021). The basic concept of SEC-MS is based on the premise that the identification of protein interactions can be determined by analyzing and comparing the elution patterns of individual proteins during SEC fractionation. When two or more proteins coelute, it indicates that there is a protein interaction or complex. However, this evidence needs to be further confirmed by applying statistical filtering and incorporating additional orthogonal information to confirm that the proteins may interact. SEC-MS is frequently used for the analysis of both monoclonal antibodies and biopharmaceuticals (Brusotti et al. 2018; Goyon et al. 2017; Haberger et al. 2016; Murisier et al. 2022; Wehr and Rodriguez-Diaz 2005; Xu et al. 2024). Moreover, this technique can ascertain the molecular weight and purity of biomolecules, detect the presence of aggregates, and identify post-translational modifications (Kirkwood et al. 2013; Kumar et al. 2023).

For the identification of immune complexes, SEC-MS was used to profile protein ADP-ribosylation and protein complexes in LPS-stimulated macrophages (Daniels et al. 2020), to probe the effect of interactomes disassembly to caspase cleavage (Scott et al. 2017), and the structural organization of the TNF-receptor signaling complex (Ciuffa et al. 2022).

To maximize the identification of PPI with SEC-MS and to precisely quantify the proteins in each SEC fraction, researchers applied SEC-MS with SWATH/DIA mass spectrometry approach, and they optimized robust methods for sample preparation and data analysis (Bludau et al. 2020, 2023; Heusel et al. 2020; Rosenberger et al. 2020).

SEC-MS has several key benefits, including the ability to separate without causing damage, to achieve high levels of detail and to conduct extensive analyses. This technology combines the capabilities of both SEC and MS methods to provide in-depth molecular insights. Nevertheless, the method necessitates intricate sample preparation and the use of specialized, expensive equipment. In addition, SEC is restricted to the analysis of soluble compounds and is not appropriate for highly hydrophobic or insoluble substances.

## Limited proteolysis‑coupled mass spectrometry (LiP‑MS)

**Fig. 3:**
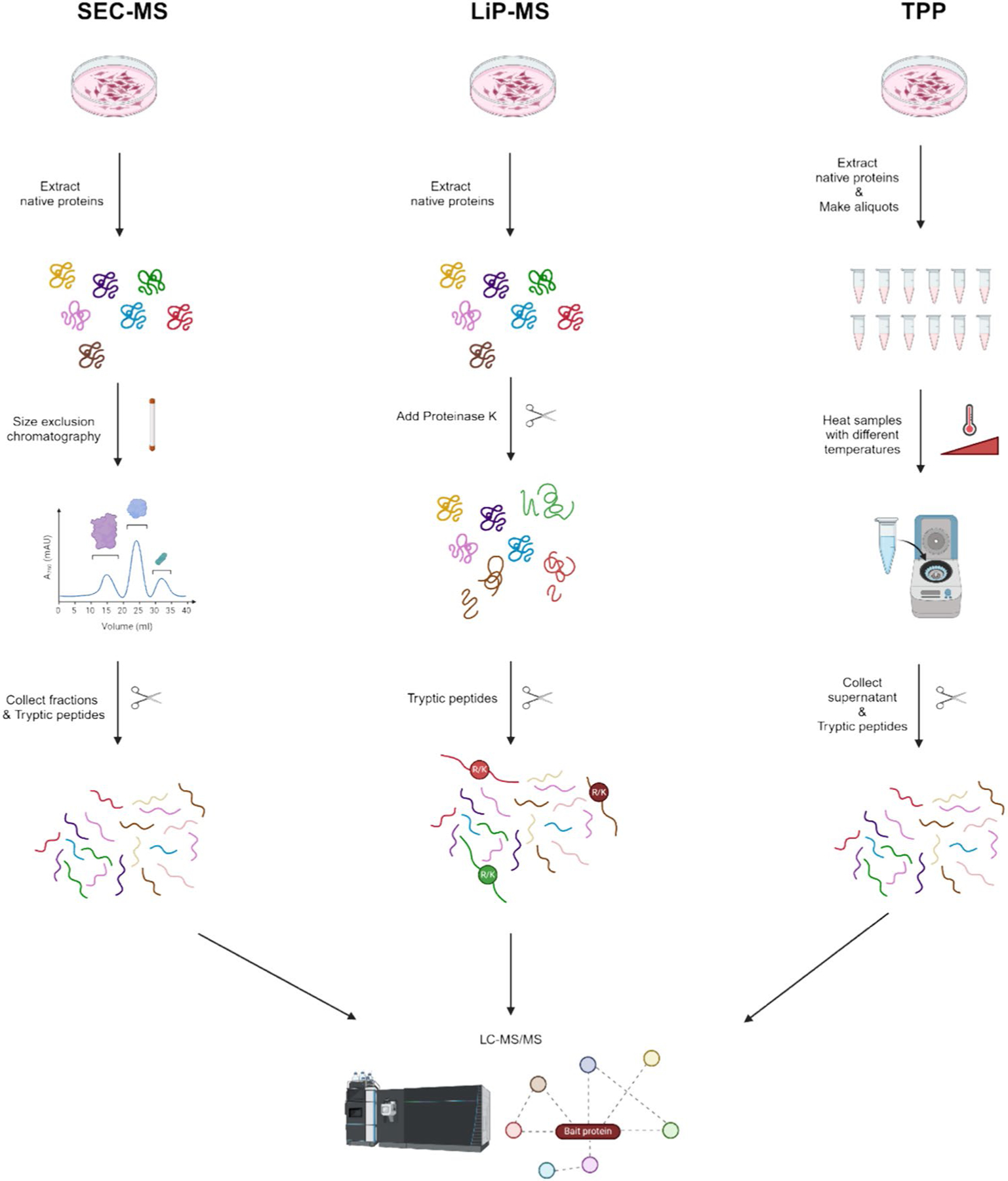
Workflows for size exclusion chromatography coupled with mass spectrometry (SEC-MS), limited proteolysis-coupled mass spectrometry (LiP-MS) and thermal proteome profiling (TPP)

LiP-MS is a cutting-edge method that has become an effective tool for understanding the intricacies of protein structural changes and interactions in biological systems. This technique depends on the specific digestion of proteins in their native conditions, then using mass spectrometry to analyze the fragments and identify any structural changes. Proteins in the LiP-MS experimental setup undergo digestion with a non-specific protease, usually proteinase K, for a short time. This enzyme cleaves peptide bonds at non-specific protease-binding sites, producing a variety of peptide fragments. Proteolysis is controlled by limiting the digestion of proteins to the protein surface. This regulated digestion offers information on the protein’s structure and conformational changes. The samples are digested with trypsin, producing semi-tryptic and tryptic peptide fragments that exhibit trypsin cleavage sites (Fig. 3). The fragments are injected into an LC–MS/MS system to identify protein conformational changes and understand complex interactions in the biological environment (Malinovska et al. 2023; Schopper et al. 2017).

For successful LiP-MS experiments, high-resolution mass spectrometry instrumentation is required to accurately analyze proteolytic fragments and their cleavage sites. Complex mass spectra interpretation and peptide sequence assignment require advanced bioinformatics algorithms that can efficiently and accurately handle large datasets.

LiP-MS provides unparalleled insights into the dynamic characteristics of proteins in biological systems. This approach provides important insights into protein structural changes and enhances comprehension of complex biological systems. LiP-MS is being used more often to study changes in protein structure caused by interactions with small molecules (Kim et al. 2023; Pepelnjak et al. 2020; Piazza et al. 2018; Sztacho et al. 2021) and disease progression (Liu and Fitzgerald 2016; Mackmull et al. 2022; Shuken et al. 2022).

LiP-MS provides notable benefits for investigating protein structural alterations and interactions, although it also has certain limitations. Using a non-specific protease such as proteinase K for initial cleavage may result in some level of protease specificity, which could cause inadequate digestion or biased cleavage patterns that mask specific protein sequences or interactions. LiP-MS is most efficient when used with pure protein samples. However, in complex biological mixtures like cell lysates or tissue extracts, the presence of many proteins and contaminants makes data interpretation difficult and requires thorough sample preparation and purification. LiP-MS may not be appropriate for insoluble proteins, particularly membrane proteins. LiP-MS offers useful insights into protein conformational changes and interactions, but its structural resolution is inherently limited when compared to techniques such as X-ray crystallography or nuclear magnetic resonance spectroscopy. LiP-MS acts at the peptide level, which makes it difficult to correctly determine protein structures or identify small conformational changes.

## Thermal proteome profiling (TPP)

TPP is based on the cellular thermal shift assay (CETSA), which measures the thermal stability of proteins in intact cells or lysates by assessing their susceptibility to thermal denaturation and aggregation (Savitski et al. 2014). Although TPP is technically more demanding, it allows for the identification of multiple targets without prior knowledge. In TPP experiments, cells or lysates are subjected to a temperature gradient, typically ranging from ambient temperature to near the denaturation temperature of most proteins. Following heat treatment, samples are rapidly cooled to halt further denaturation and then lysed to release soluble proteins (Fig. 3). The soluble proteome is then analyzed by mass spectrometry to quantify changes in protein abundance across different temperature conditions (Becher et al. 2016a, b; Franken et al. 2015; Reinhard et al. 2015).

To enable quantitative analysis, TPP experiments often employ isobaric labeling techniques such as tandem mass tags or stable isotope labeling by amino acids in cell culture. Isobaric labeling allows for multiplexed quantification of protein abundance across multiple temperature conditions within a single mass spectrometry experiment, thereby increasing throughput and reducing experimental variability.

A multitude of experimental procedures can be conducted utilizing TPP. Cells are heated to multiple temperatures when a single compound concentration is applied, and this procedure is referred to as the temperature range TPP (TPP-TR) experiment. This method allows for the identification of the majority of targets of a compound present in cell extracts, demonstrating a reproducible change in melting temperature of above 1 °C. There was little association between the Tm shift and the compound’s affinity for each binding protein. The degree of thermal stabilization is influenced by both the ligand’s affinity and the melting thermodynamics of the native protein. A compound concentration range for TPP (TPP-CCR) can be used to calculate affinity estimates through TPP. During TPP-CCR, cells are exposed to various concentrations of a compound and then subjected to a uniform temperature. Finally, a two-dimensional TPP (2D-TPP) method including incubating cells with various chemical concentrations and subjecting them to different temperatures has become accessible (Becher et al. 2016a, b; Mateus et al. 2020). This expansion enables a quick assessment of compound affinity towards the target and is far more effective at pinpointing targets.

TPP has been used in the innate immune system to study structural changes in heat shock proteins of activated macrophages, in combination with LiP revealing that the structures of molecular chaperones such as HSP60 can vary greatly during macrophage activation (Zhang et al. 2021). It enabled identification of antiviral inhibitor targets with broad range of activity against RNA viruses (Tampere et al. 2020), and analysis of protein complex formation and dissociation during human cytomegalovirus infection. (Hashimoto et al. 2020; Justice et al. 2021). These investigations have shown that protein–ligand interactions are altered depending on temperature, offering an understanding of the molecular processes involved in innate immune responses.

The TPP has numerous benefits for investigating protein stability and interactions. This method provides a comprehensive way to analyze protein–ligand interactions and complex formation in reaction to thermal stress across the whole proteome. TPP experiments can be conducted in intact cells or lysates to analyze protein stability under physiological conditions.

Nevertheless, TPP has drawbacks such as the possibility of non-specific protein aggregation and the requirement for specialized mass spectrometry instrumentation and skills in proteomics and bioinformatics. TPP experiments may be prone to artifacts and false-positive identifications, requiring thorough validation and controls to guarantee result reliability.

## Data analysis and visualization

Using these five methodologies, we can acquire valuable insights into protein complexes. In terms of MS/MS peptides quantification, MaxQuant (Cox and Mann 2008) and Proteome Discoverer are widely used. In addition, in-house developed R or Python packages, such as MSstatsLiP (Malinovska et al. 2023), Rtpca (Kurzawa et al. 2021), NPARC (Childs et al. 2019), or Tapioca (Reed et al. 2024), can be used according to their respective objectives. To characterize protein networks, PPI databases (e.g., BioGRID (Oughtred et al. 2021), Reactome (Milacic et al. 2024), STRING (Szklarczyk et al. 2023), CORUM (Giurgiu et al. 2019) and IntAct (Del Toro et al. 2022)) that suggest a complex interaction are commonly used. The program known as Cytoscape is extensively employed to analyze expression data and genetic interactions, as well as PPIs, and for visualization of biological networks (Shannon et al. 2003).

The purpose of functional annotation is to gather details regarding the established biological function of PPIs. Gene Ontology (GO) is most often used for annotation, offering an extensive set of terms, which, however, might lead to an overwhelming number of terms connected with a certain protein (Ashburner et al. 2000).

The purpose of enrichment analysis is to ascertain if a portion of the network is enriched in a related function. After adding GO functional annotation to the network, enrichment analysis can be performed on each of the bait protein’s interaction partners. If the proteins exhibit a greater probability of sharing a certain set of GO terms than what would be anticipated by a random assignment of all terms in the network, it is probable that these proteins are implicated in the corresponding biological process or cellular function, dependent on the branch of GO. Ontology-based enrichment analysis can be conducted using many tools, such as web-based platforms such as DAVID (Sherman et al. 2022) and several Cytoscape applications including BiNGO (Maere et al. 2005), ClueGO (Bindea et al. 2009), NOA (Zhang et al. 2013) and ReactomeFIViz (Wu et al. 2014).

## Conclusion and perspective

In this review, we presented various mass spectrometry-based techniques for characterizing PPIs and their applications to understanding biological processes (Table 1).

Through the exploration of these techniques, it is evident that mass spectrometry has become an indispensable tool for unraveling the complexities of molecular interactions within biological systems. From elucidating signaling cascades triggered by endotoxins to identifying drug-target interactions, the ability to characterize PPIs with high sensitivity and specificity has greatly enhanced our understanding of fundamental biological processes.

Looking ahead, continued advancements in mass spectrometry instrumentation, data analysis algorithms, and sample preparation methodologies hold promise for further expanding the capabilities of PPI characterization techniques. Integration with other omics approaches such as genomics, transcriptomics, and metabolomics will enable a more holistic understanding of cellular processes and disease mechanisms.

Moreover, the development of multiplexed and high-throughput screening methods will facilitate the rapid and comprehensive analysis of PPI networks under diverse conditions. These advancements will not only deepen our understanding of basic biology but also pave the way for the discovery of novel therapeutic targets and the development of precision medicine strategies.

## Figures and Tables

**Table 1:** Advantages and disadvantages of mass spectrometry-based approaches

Technology	Advantages	Disadvantages
Affinity purification mass spectrometry (AP-MS)	High specificity Quantitative analysis Well-established method	Limited to known interactors Requirement for specific reagents Potential for non-specific binding
Proximity labeling mass spectrometry (PL-MS)	Captures protein–protein interactions Enables spatial and temporal proteomic analysis Compatible with diverse experimental systems	Risk of false positives Background noise Optimization challenges
Cross-linking mass spectrometry (XL-MS)	Captures transient protein–protein interactions Provides structural insights Allows identification of interacting residues	Complex data interpretation Requires cross-linker optimization Sensitivity to cross-linker concentration
Size exclusion chromatography mass spectrometry (SEC-MS)	Separates molecules based on their size Can be applied to various types of samples Does not denature proteins or alter the structure	Lower resolution compared to HPLC Some samples may adsorb to the column material Complex data interpretation
Limited proteolysis-coupled mass spectrometry (LiP-MS)	Provides structural information Reveals protein conformational changes Detects protein–protein interactions	Limited to proteins amenable to proteolysis Requires expertise in data interpretation Not suitable for all protein complexes
Thermal proteome profiling (TPP)	Identifies protein–ligand interactions Measures protein stability changes High throughput	Limited to soluble proteins Requires stringent control experiments May miss low abundant or transient proteins

## Data Availability

No datasets were generated or analysed during the current study.
